# Molecular Composition and Ligand Binding Characteristics of Native Ionotropic GABA Receptors in Rice Stem Borer, *Chilo suppressalis*

**DOI:** 10.3390/insects17050477

**Published:** 2026-05-06

**Authors:** Enling Zhan, Jie Luo, Yuqing Zhang, Junyan Wang, Shuang Ni, Chunqing Zhao

**Affiliations:** 1 Key Laboratory of Integrated Pest Management on Crops in East China, Ministry of Agriculture and Rural Affair, College of Plant Protection, Nanjing Agricultural University, Nanjing 211800, China; eileenzhan0904@163.com (E.Z.); 2024802298@stu.njau.edu.cn (J.L.); 2023802241@stu.njau.edu.cn (Y.Z.); 18952802515@163.com (J.W.); 2 Plant Protection, Quarantine and Cultivated Land & Fertilizer Management Station, Huzhou Municipal Bureau of Agriculture and Rural Affairs, Huzhou 313000, China; 19857183372@163.com; 3 State Key Laboratory of Agricultural and Forestry Biosecurity, College of Plant Protection, Nanjing Agricultural University, Nanjing 211800, China

**Keywords:** native iGABAR subunits, N-terminal truncation, transcripts, postsynaptic membrane, desmethyl-broflanilide

## Abstract

The ionotropic γ-aminobutyric acid receptor (iGABAR) is an important insecticidal molecular target. However, the native iGABARs composition remains unknown in insect. Here, *Cs*Rdl1, truncated transcripts of *Cs*Rdl2 and *Cs*Lcch3 were obtained in the rice stem borer (RSB), *Chilo suppressalis* Walker. The N-terminal-truncated *Cs*RDL2 (∆N-*Cs*RDL2) and N-terminal-truncated *Cs*LCCH3 (∆N-*Cs*LCCH3) were deduced and studied *in vivo*, and desmethyl-broflanilide (DMBF) binding characteristics were simulated *in silico*. Genome and transcriptome analyses revealed truncated transcripts of *Cs*Rdl2 and *Cs*Lcch3 encoded 48 kDa of ∆N-*Cs*RDL2 and 37 kDa of ∆N-*Cs*LCCH3, respectively. The *Cs*RDL1, *Cs*RDL2 and *Cs*LCCH3 were detected respectively from native iGABARs at molecular weights (Mws) ≥ 440 kDa in BN-PAGE. In BN/SDS-PAGE, three *Cs*RDL1 bands (~54, ~55 and ~70 kDa), one *Cs*RDL2 band (~48 kDa) and one *Cs*LCCH3 band (~37 kDa) were identified in native iGABARs at Mws ≥ 669 kDa, and corresponded to *Cs*RDL1ad, *Cs*RDL1bd, post-translationally modified *Cs*RDL1, ∆N-*Cs*RDL2 and ∆N-*Cs*LCCH3, respectively. Immunofluorescence confirmed these three subunits distributed in the same region of adult heads. Finally, DMBF displayed higher binding affinities for heteromeric iGABARs than for homomeric *Cs*RDL1 iGABAR *in silico*. These findings confirm that ∆N-*Cs*RDL2 and ∆N-*Cs*LCCH3 in native iGABARs might support the rational design of novel insecticides.

## Simple Summary

Ionotropic γ-aminobutyric acid receptor (iGABAR) is one of the key molecular targets for developing insecticides. However, the molecular composition and ligand binding characteristics of native iGABARs have not been characterized in insects. In this study, native iGABARs composed of *Cs*RDL1, *Cs*RDL2 or *Cs*LCCH3 were identified from postsynaptic membranes of rice stem borer (RSB). N-terminal-truncated transcripts and proteins of *Cs*RDL2 (∆N-*Cs*RDL2) and *Cs*LCCH3 (∆N-CsLCCH3), as well as *Cs*RDL1ad, *Cs*RDL1bd and post-translationally modified *Cs*RDL1, were detected in native iGABARs. In addition, *Cs*RDL1, *Cs*RDL2 and *Cs*LCCH3 were distributed in the same region in the adult head. Notably, the binding affinities of desmethyl-broflanilide (DMBF) to heteromeric iGABARs assembled by *Cs*RDL1, ∆N-*Cs*RDL2, and ∆N-*Cs*LCCH3 were higher than those of DMBF to homomeric iGABAR assembled by *Cs*RDL1. Overall, our findings indicated that ∆N-*Cs*RDL2 and ∆N-*Cs*LCCH3 could compose native iGABARs with *Cs*RDL1, and they attenuate DMBF binding to iGABARs in the RSB. These results provide new insights into the molecular constituents of postsynaptic ion channels and support the rational design of novel insecticides.

## 1. Introduction

γ-aminobutyric acid (GABA) is the major inhibitory neurotransmitter in the nervous systems of both vertebrates and invertebrates, and mediates fast inhibitory synaptic transmission by acting on ionotropic GABA receptors (iGABARs) [1]. As a member of the cys-loop ligand-gated ion channel (LGIC) superfamily, iGABARs have been identified as one of the major molecular targets for drugs and insecticides. Notably, the pharmacological properties of insect iGABARs exhibit significant differences to those of vertebrate iGABARs, which provide a foundation for developing insect-specific insecticides [2]. The structural and functional diversity of iGABARs is attributed to the varied composition of subunit complexes within the LGIC superfamily [3]. In vertebrates, the pore-forming subunits of native iGABARs have been isolated and characterized [4], and are assembled by five subunits *in vivo* [5,6]. For instance, iGABARs containing 2α1/2β/γ2 subunits localize at synapses in the cerebellar granule cells [7]. Furthermore, 12 native subunit assemblies and their corresponding three-dimensional (3D) structures of iGABARs have been precisely defined [6]. Additionally, alternative splicing of pre-mRNA has been recognized as a key mechanism for generating iGABAR diversity [8]. Compared with its N-terminal-truncated transcript, the completed γ2 subunit reduces the cell surface expression of α1β3γ2 receptors by 50% [9]. Moreover, alternative splicing of the mouse *Mus musculus* Linnaeus α4 subunit results in an N-terminal-truncated α4 (∆N-α4), which selectively attenuates GABA-induced currents [10].

In insects, the predicted subunits of iGABARs include RDL (resistant to dieldrin), LCCH3 (ligand-gated chloride channel homolog 3), GRD (GABA and glycine receptor-like subunit of *Drosophila*), and CG8916 [2,11,12,13,14]. *In vitro* experimental data have demonstrated that the functional diversity of homomeric or heteromeric iGABARs can be revealed through a heterogeneous expression system. For example, compared with homomeric iGABAR assembled by RDL subunits, the sensitivity of heteromeric iGABARs assembled by RDL and LCCH3 to bicuculline and picrotoxin is enhanced and reduced, respectively [15]. Furthermore, RDL and LCCH3 of human louse *Pediculus humanus humanus* Linnaeus reconstitute functional heteromeric iGABARs in the *Xenopus* oocyte heterologous expression system, which respond to GABA in a concentration-dependent manner and are sensitive to picrotoxin and fipronil [16]. Moreover, post-transcriptional modifications of messenger RNA (mRNA), including alternative splicing and RNA editing, can increase RDL heterogeneity [17]. Two transcript variants of the Rdl, designated as Rdlad and Rdlbd, arose from alternative splicing events at exons 3 and 6 and were identified [1]. Truncated transcripts of Rdl1 and Lcch3 exist in the silkworm *Bombyx mori* Linnaeus [18]. Alternative splicing events in CsRdl of the rice stem borer (RSB) and the splicing factor Nova have also been reported [19]. However, the native truncated isoforms of iGABAR subunits generated by alternative splicing have not yet been identified in the RSB.

Insect iGABARs are important insecticidal molecular targets [20,21]. Currently, insecticides acting on iGABARs, such as fipronil, avermectin and broflanilide, are widely applied in the control of Lepidoptera pests in agriculture [20,21,22]. As a *meta*-diamide insecticide, broflanilide has been reported to be metabolized to desmethyl-broflanilide (DMBF) in insects and then embedded into the cavity between the transmembrane domain 1 (TM1) and TM3 of RDL [23]. Furthermore, glycine at the third position (G3’) in TM3 of RDL is associated with the sensitivity of insects to DMBF [23,24]. In recent, 3D structures of iGABAR assembled by honeybee *Apis mellifera* L. RDL have been precisely defined, and the binding pocket and key residue interacting with abamectin, fluralaner, fluxametamide, and isocycloseram, in iGABAR have been reported [25]. As is well-known, the RSB is an important Lepidoptera pest in Asia, especially in China [26]. Therefore, the native iGABARs of RSB *in vivo*, and DMBF *in silico* binding to iGABARs assembled by *Cs*RDL1, *Cs*RDL2 and CsLCCH3 were studied in the present study. These results will provide evidence for alternative splicing events and facilitate the development of targeted insecticides.

## 2. Materials and Methods

### 2.1. CsRdl1, CsRdl2 and CsLcch3 Transcripts Analyzation

The genome of RSB (InsectBase ID: IBG_00177) [27] and its transcriptome (InsectBase ID: PRJNA522832) were used to explore the transcripts of *Cs*Rdl1, *Cs*Rdl2 and *Cs*Lcch3. The nucleotide sequences of *Cs*Rdl1ad (GenBank accession no. KX856969.1), *Cs*Rdl1bd (GenBank accession no. KX856966.1), *Cs*Rdl2 (GenBank accession no. KX856970.1) and *Cs*Lcch3 (GenBank accession no. KX856968.1) were were aligned against the RSB genome using BLASTN v2.17.0+ (National Center for Biotechnology Information (NCBI), Bethesda, MD, USA) with an e-value threshold of 1e^−5^ to map their genomic locations. Subsequently, based on the annotations of the genomic GFF3 file, GffRead v0.12.7 (Center for Computational Biology, Johns Hopkins University, Baltimore, MD, USA) was used to extract the corresponding transcript sequences from the RSB genome by matching the transcript coordinates in the GFF3 file with the genomic sequence and combining the genomic location information of the target genes. Raw transcriptomic reads were mapped to the reference genome via HISAT2 v2.2.1 (Center for Computational Biology, Johns Hopkins University, Baltimore, MD, USA) [28]. The alignment outputs were processed with Samtools v1.21 (Wellcome Sanger Institute, Cambridge, UK), and the resulting sorted BAM file was then used for transcript assembly and GTF file generation using StringTie v2.2.1 (Johns Hopkins University, Baltimore, MD, USA). The resulting GTF file was applied to retrieve transcript sequences from the genome with GffRead v0.12.7. Transcript sequences were extracted using BEDTools software v2.31.1 (Quinlan Laboratory, University of Utah, Salt Lake City, UT, USA) [29], and transcript profiles were visualized using the IGV browser [30]. The signal peptide was analyzed in the SignalP v5.0 server (https://services.healthtech.dtu.dk/services/SignalP-5.0/) (Center for Biological Sequence Analysis, Technical University of Denmark, Kongens Lyngby, Denmark). The protein domains were analyzed in the SMART server v10 (https://smart.embl.de/) (European Molecular Biology Laboratory (EMBL), Heidelberg, Germany).

### 2.2. Insect Rearing and Antibodies Synthesis

Larvae of RSB were collected from Huzhou city (Zhejiang province, China) and reared in an artificial climate incubator at 27 ± 1 °C, with a 16: 8 h light/dark photoperiod and 60–80% relative humidity. Larvae and adults were fed respectively with wild rice stem *Zizania latifolia* (Griseb.) Hance ex F.Muell. and 10% (*v*/*v*) honey water as a nutritional supplement [31]. Peptides of *Cs*RDL1 (DPHTLSKMGTIGRC), *Cs*RDL2 (PLPPPRTSTLNRPLC) and *Cs*LCCH3 (AKLKNRDQMSTSTSC), which located at the intracellular cytoplasmic loop between TM3 and TM4, were used as antigens, and GenScript (Nanjing, China) was commissioned to synthesize rabbit polyclonal antibodies against these peptides [32].

### 2.3. Postsynaptic Membrane Extraction

The postsynaptic membrane was extracted from the head and thorax of the RSB adults as described by Morató et al. (2017) [33]. In detail, 0.35 g head or thorax from the RSB adults were homogenized with 1 mL isolation buffer (0.32 M sucrose, 0.1 mM CaCl_2_ and 0.1 mM MgCl_2_, pH 7.4) and solubilized with 6 mL 2 M sucrose and 2.5 mL 0.1 mM CaCl_2_ at 4 °C. The solution was transferred into 38.5 mL centrifuge tubes, and 2.5 mL of 1 M sucrose/0.1 mM CaCl_2_ solution was slowly layered on top of each tube to form a sucrose gradient sequentially. Subsequently, centrifugation was performed at 100,000× *g* for 3 h at 4 °C using a swinging-bucket rotor centrifuge. The white ring at the interphase between 1.25 M and 1 M sucrose was carefully collected, diluted with nine volumes of isolation buffer, and then centrifuged at 15,000× *g* for 30 min at 4 °C. The resulting pellet was recovered and resuspended in 1 mL isolation buffer. The suspended solution was diluted in 5 mL of 0.1 mM CaCl_2_, followed by incubation with 5 mL of ice-cold 2 × Solubilization Buffer (40 mM Tris with 2% Triton X-100, pH 6.0) for 50 min on ice under high agitation. After incubation, centrifugation was conducted at 40,000× *g* for 30 min at 4 °C, and the pellet was recovered, washed with 2 mL of 1 × Solubilization Buffer I (20 mM Tris with 1% Triton X-100, pH 6.0) and subsequently incubated in 10 mL of ice-cold 1 × Solubilization Buffer II (20 mM Tris with 1% Triton X-100, pH 8.0) for 50 min on ice under high agitation. After incubation, centrifugation was performed at 40,000× *g* for 30 min at 4 °C, and the pellet corresponds to the postsynaptic fraction was harvested. 

### 2.4. 1-DE Gel Electrophoresis: Blue Native-PAGE (BN-PAGE)

BN-PAGE was performed as described previously [34]. In brief, the postsynaptic fraction was solubilized with 1% Lauryl Maltose-neopentyl glycol (MNG) (809897, MedChemExpress, Shanghai, China) in 40 mM Tris-Cl buffer (pH 8.0) with proteinase inhibitor (Roche, Basel, Switzerland) [35]. The postsynaptic membrane proteins (50 μg) were seperated by BN-PAGE gel (3.5% stacking and 4–12% separating gel). Electrophoresis was performed using the anode buffer (25 mM imidazole, pH 7.0) and cathode buffer B (pH 7.0) containing 50 mM tricine, 7.5 mM imidazole, and 0.02% *w*/*v* Coomassie blue G-250. The voltage was set to 100 V for 30 min until the samples entered the separating gel, and then set to 140 V until the blue running front had moved ~1/3 desired total running distance. Subsequently, the cathode buffer B was replaced with cathode buffer B/10 (pH 7.0) containing 50 mM tricine, 7.5 mM imidazole, and 0.002% *w*/*v* Coomassie blue G-250, and electrophoresis continued until the dye front reached the bottom of the gel. Molecular weights (Mws) were determined using the high-molecular-weight calibration kit for native electrophoresis (GE Healthcare, Marlborough, MA, USA). The gel was stained with Coomassie Brilliant Blue R-250 (CBB R-250). BN-PAGE gels were used for electroblotting of native proteins [34], and cut into gel pieces for nanoLC-MS/MS [35] (Text S1) and BN/SDS-PAGE. Immobilon^®^ Western Chemiluminescent HRP Substrate (MilliporeSigma, Burlington, MA, USA) was used for visualization. The protein marker was stained with Ponceau S staining solution (0.1% *w*/*v* Ponceau S and 5% *v*/*v* acetic acid).

### 2.5. 2-DE Gel Electrophoresis: BN/SDS-PAGE and Western Blots

It has been reported that not all antibodies can work perfectly in SDS-PAGE and BN-PAGE in the Western blot assay because the target peptide may not be exposed in a complex [36]. Thus, BN/SDS-PAGE was applied to further verify the BN-PAGE bands. The native gel lanes were equilibrated with gentle agitation for 1 h in an equilibration buffer (1% *w*/*v* SDS and 1% *v*/*v* mercaptoethanol), followed by a brief rinse with water. Each gel lane was gently placed onto an SDS-PAGE gel (5% stacking and 10% separating gel). The SDS-PAGE electrophoresis buffer contained 25 mM Tris, 192 mM glycine and 0.1% *w*/*v* SDS. The voltage was set at 100 V for 30 min until the sample had entered the gel, and then increased to 120 V until the dye front reached the bottom of the gel. Mws were determined using PageRuler Prestained Protein Ladder (Thermo Fisher Scientific, Waltham, MA, USA). The gel was stained with CBB R-250. Following electrophoresis, proteins were transferred to a PVDF membrane (MilliporeSigma, Burlington, MA, USA) for Western blot analysis. PVDF membrane was incubated with 1:1000 dilution of a primary antibody against *Cs*RDL1, *Cs*RDL2 or CsLCCH3, respectively, followed by a Mouse Anti-Rabbit IgG Antibody [HRP], mAb (GenScript) (1:20,000). Immobilon^®^ Western Chemiluminescent HRP Substrate was used for visualization.

### 2.6. 3-DE Gel Glectrophoresis: BN/SDS/SDS-PAGE

The BN/SDS/SDS-PAGE gel electrophoresis was performed according to a previously published protocol [37]. The BN/SDS-PAGE gel were cut into individual gel lanes and equilibrated in an equilibration buffer for 30 min. Then, the gel lanes were rinsed with water, and each gel lane was placed onto an SDS-PAGE gel (5% stacking and 10% separating gel). The SDS-PAGE electrophoresis was performed as abovemented procedures, except the voltage was set at 80 V for 30 min at the initial stage. Meanwhile, the western blot analysis was subsequently performed in accordance with the above mentioned procedures.

### 2.7. Immunofluorescence

The adult RSB heads were removed carefully and immediately embedded in O.C.T. embedding compound (Sakura Finetek, Tokyo, Japan). Cryosections of head were cut at 10 μm and directly melted onto glass microscope slides. Three successive cryosections were collected for subunit localization, blank control, and negative control, respectively. Sections were firstly fixed in 4% paraformaldehyde for 20 min at room temperature, washed with PBS buffer (pH 7.4), and then incubated with methanol for 5 min at −20 °C to permeabilize the membrane. Cryosections of heads were stained with 1:200 dilutions of a primary antibody against *Cs*RDL1, *Cs*RDL2 or CsLCCH3, respectively, after being blocked with PBS buffer containing 3% *w*/*v* BSA and 10% *v*/*v* goat serum (Solarbio, Beijing, China), and then stained with a 1:1000 dilution of Goat Anti-Rabbit IgG H&L (Alexa Fluor^®^ 488) preadsorbed (ab150081, Abcam, Cambridge, UK). The PBS buffer (3% *w*/*v* BSA and 10% *v*/*v* goat serum) without primary antibody was used as the blank control. Rabbit pre-immune serum was used as a primary antibody in the negative control. A Zeiss inverted fluorescence microscope was used for observation. The different brain regions were named according to *Drosophila melanogaster* Meigen adult brains [38].

### 2.8. Molecular Docking Analysis

Molecular docking was performed to evaluate the binding affinity between iGABAR complex and DMBF. First, the 3D structures of iGABAR models were predicted using AlphaFold 3 based on the protein sequences of *Cs*RDL1bd (GenBank accession no. ASY91958.1), ∆N-*Cs*RDL2, and ∆N-*Cs*LCCH3 (Table S2), and the structural quality assessment studies were performed using ERRAT and PROCHECK [39,40]. The structure of DMBF were retrieved from the PubChem database (https://pubchem.ncbi.nlm.nih.gov/) (NCBI) and ChemDraw 20.0 [41]. The binding pockets of iGABAR models were predicted by the CASTp v3.0 server (http://sts.bioe.uic.edu/castp/) (University of Illinois at Chicago, Chicago, IL, USA) [42]. AutoDock Vina Tools v1.5.7 (Center for Computational Structural Biology (CCSB), The Scripps Research, La Jolla, CA, USA) [43] was utilized to preprocess the iGABAR models and DMBF. This process included the removal of water molecules, the addition of hydrogen atoms, and the calculation of charges to generate the required input files. Subsequently, a docking grid was set around G3’ in TM3 of RDL in complexes, and the grid center was designated at dimension (x, y, and z) (Table S4). Molecular docking simulations were conducted using AutoDock Vina Tools v1.5.7. Finally, docking poses exhibiting optimal binding affinity and reasonable conformations were selected and visualized using PyMOL v3.1.6.1 (Schrödinger, LLC, New York, NY, USA) and the specific interaction modes between the ligands and protein residues were analyzed using BIOVIA Discovery Studio 2021 (Dassault Systèmes, San Diego, CA, USA).

### 2.9. Statistics

Statistical analyses were performed using GraphPad Prism 6 (GraphPad Software Inc., San Diego, CA, USA). The grayscale intensity of the band was measured with Image J v1.46r software (National Institutes of Health (NIH), Bethesda, MD, USA).

## 3. Results

### 3.1. Transcripts of CsRdl1, CsRdl2 and CsLcch3 in the Genome and Transcriptome of RSB

The transcripts of *Cs*Rdl1, *Cs*Rdl2 and *Cs*Lcch*3* were obtained from the RSB genome and transcriptome. The transcripts (Csup004269.1 and STRG.8675.1) corresponded to *Cs*Rdl1bd were identified respectively from genome and transcriptome (Figure S1 and Table 1) and its encoding amino acid sequences were same as *Cs*RDL1bd (GenBank accession no. ASY91958.1) (Figure S2). The transcripts (Csup004319.1 and STRG.8682.1) were encoded ∆N-*Cs*RDL2 (Figure S3 and Table 1), which lacks 72-amino acid at the N-terminus, including signal peptide, and shared 85.48% similarity with *Cs*RDL2 (GenBank accession no. ASY91962.1) (Figure S4). Three transcripts of *Cs*Lcch3 (Csup009529.1, STRG.622.1 and STRG.622.2) were obtained (Figure S5 and Table 1) and their encoded amino acids shared 100%, 66.67% and 67.07% similarity with *Cs*LCCH3 (GenBank accession no. ASY91960.1), respectively. Thereinto, the transcripts (STRG.622.1 and STRG.622.2) were encoded ∆N-*Cs*LCCH3, which lacks 162-amino acid at the N-terminus, including signal peptide and three loops (A, D and E) (Figure S6). 

### 3.2. Identified Native iGABAR Complexes by Antibodies 

**Figure 1:**
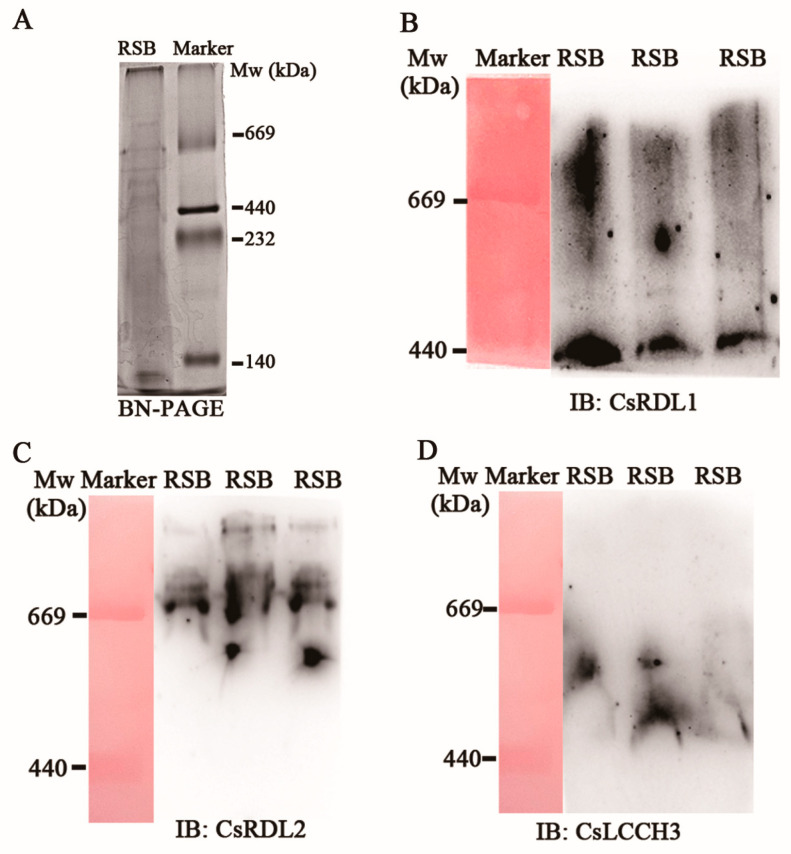
Separation of the native iGABAR complexes via BN-PAGE. (**A**) Proteins were stained with CBB R-250. iGABAR complexes identified with antibodies against *Cs*RDL1 (**B**), *Cs*RDL2 (**C**) or *Cs*LCCH3 (**D**), respectively. Marker was stained with Ponceau S staining solution in red/purple bands. IB, Immunoblot.

BN-PAGE was used to separate the native iGABARs complexes from the postsynaptic membrane proteins of RSB. Multiple bands were observed at Mws ≥ 440 kDa (Figure 1A). Antibodies against *Cs*RDL1, *Cs*RDL2 and *Cs*LCCH3 were respectively used to identify iGABARs at Mws ≥ 440 kDa. Two bands (440 kDa and Mws > 440 kDa) (Figure 1B), four bands (one bands at 440 kDa < Mws < 669 kDa, one bands at 669 kDa and two bands at Mws > 669 kDa) (Figure 1C) and one band (at 440 kDa < Mws < 669 kDa) (Figure 1D) were identified by the antibodies against *Cs*RDL1, *Cs*RDL2 and *Cs*LCCH3, respectively. Subsequently, the gel pieces with the bands at Mws ≥ 440 kDa were excised and analyzed by nanoLC-MS/MS. Unexpectedly, only the peptides and corresponding abundance of *Cs*RDL2 was identified (Table S1).

### 3.3. Identified CsRDL1, ∆N-CsRDL2 and ∆N-CsLCCH3 from Native iGABARs

**Figure 2:**
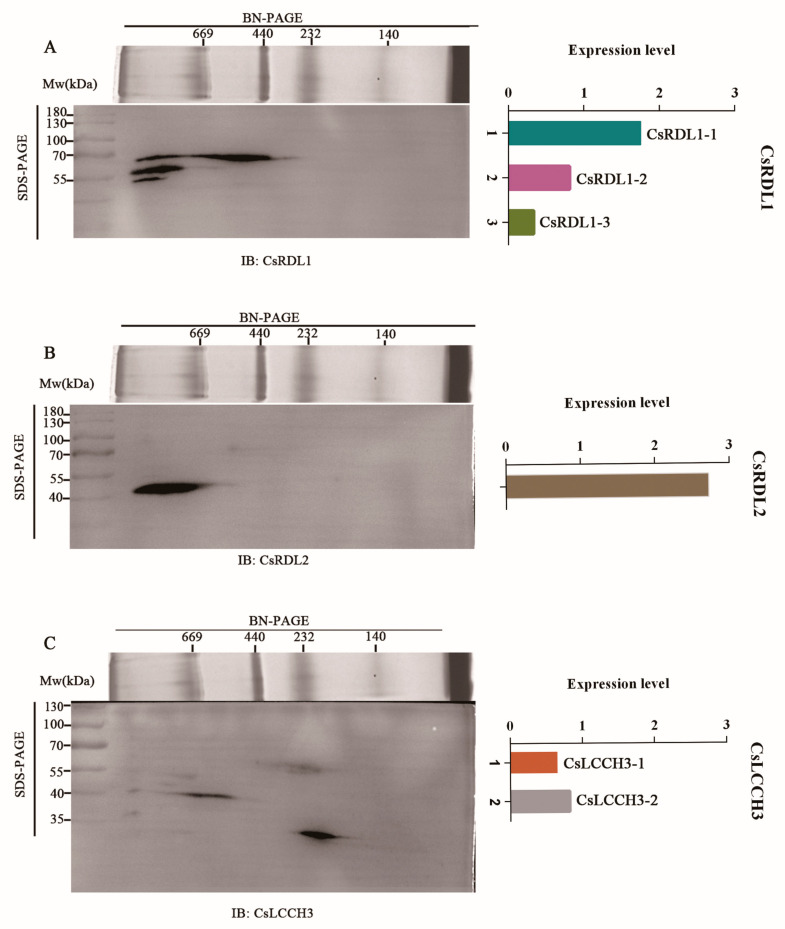
Separation of subunits in the native iGABARs via BN/SDS-PAGE. (**A**) *Cs*RDL1; (**B**) *Cs*RDL2; (**C**) *Cs*LCCH3.

Multiple bands were observed in BN/SDS-PAGE (Figure S7). Three bands with distinct Mws (~54 kDa, ~55 kDa and ~70 kDa) were detected by the antibody against *Cs*RDL1, and defined as *Cs*RDL1-3 isoform, *Cs*RDL1-2 isoform and *Cs*RDL1-1 isoform, respectively (Figure 2A). Thereinto, the expression level of three isoforms in descending order in *Cs*RDL1-1, *Cs*RDL1-2, and *Cs*RDL1-3, respectively (Figure 2A). *Cs*RDL2 exhibited a single, highly intense band at ~48 kDa (Figure 2B). The expression level of *Cs*RDL2 was higher than that of *Cs*RDL1-1 (Figure 2B). Two scattered bands with distinct Mws (~56 kDa and ~37 kDa) were detected by the antibody against *Cs*LCCH3 and defined as *Cs*LCCH3-1 isoform and *Cs*LCCH3-2 isoform, respectively (Figure 2C). Thereinto, the expression level of two isoforms in ascending order in *Cs*LCCH3-1 and *Cs*LCCH3-2, respectively (Figure 2C).

The Mws of iGABARs were divided into three ranges: Mws ≥ 669 kDa, 440 kDa ≤ Mws < 669 kDa and Mws < 440 kDa, and the composition and ratio of each subunit of native iGABARs was speculated based on the expression levels of subunits (*Cs*RDL1-1, *Cs*RDL1-2, *Cs*RDL1-3, *Cs*RDL2, *Cs*LCCH3-1, and *Cs*LCCH3-2) in each range (Figure S8). Furthermore, the BN/SDS-PAGE gel was divided into four gel lanes according to *Cs*RDL2 immunoblot for 3-DE gel electrophoresis (Figure S9A). The spots of *Cs*RDL2 and *Cs*LCCH3-2 in 3-DE gel electrophoresis were observed at 48 and 37 kDa, respectively, which have equal Mws with 2-DE gel electrophoresis (Figure S9B).

### 3.4. Distribution of CsRDL1, CsRDL2 and CsLCCH3 in the Adult RSB Head 

**Figure 3:**
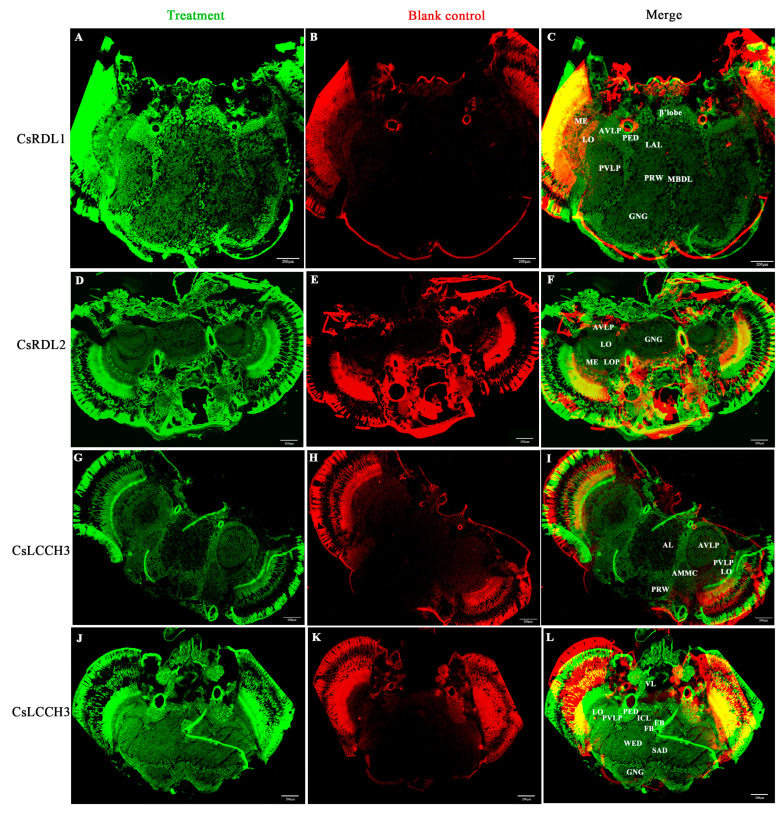
Distribution of *Cs*RDL1, *Cs*RDL2 and *Cs*LCCH3 in the adult RSB head. (**A**) *Cs*RDL1; (**D**) *Cs*RDL2; (**G**,**J**) *Cs*LCCH3; (**B**,**E**,**H**,**K**) blank controls; (**C**,**F**,**I**,**L**) merged images. AL, antennal lobe; AMMC, antennal mechanosensory and motor center; AVLP, anterior ventrolateral protocerebrum; EB, ellipsoid body; FB, fan-shaped body; GNG, gnathal ganglia; ICL, interior clamp; LAL, lateral accessory lobe; LO, lobula; LOP, lobula plate; MBDL, median bundle; ME, medulla; PED, pedunculus; PRW, prow; PVLP, posterior ventrolateral protocerebrum; SAD, saddle; VL, vertical lobe; and WED, wedge; β’lobe, medial lobe division in mushroom body. Scale bar, 200 μm. The green channel corresponds to signals from the treatment group, while the red channel represents signals from the blank control group.

The distribution of *Cs*RDL1, *Cs*RDL2 and *Cs*LCCH3 in the adult RSB head was respectively explored in tissue-cryosections. Compared with the blank control, *Cs*RDL1 was detected in the β’lobe, median bundle (MBDL), anterior ventrolateral protocerebrum (AVLP), lobula (LO) and the periphery of the posterior ventrolateral protocerebrum (PVLP) and gnathal ganglia (GNG) (Figure 3A–C). *Cs*RDL2 was distributed in the AVLP, LO, lobula plate (LOP) and the periphery of the GNG (Figure 3D–F). *Cs*LCCH3 was distributed in the AVLP, ellipsoid body (EB), PVLP, pedunculus (PED), LO, vertical lobe (VL) and the periphery of the GNG, antennal lobe (AL), prow (PRW) and antennal mechanosensory and motor center (AMMC) (Figure 3G–L and Figure S10).

### 3.5. Binding Affinities of DMBF to Different Assembled iGABAR Models 

iGABAR models assembled by *Cs*RDL1bd, ∆N-*Cs*RDL2 and ∆N-*Cs*LCCH3 at different subunit ratios (5:0:0, 3:1:1, 2:2:1 and 1:3:1) were predicted using sequence-based models via AlphaFold 3. The results accessed from ERRAT (Table S3) and PROCHECK (Figure S11) confirmed that the predicted models of iGABAR have good quality.

**Figure 4:**
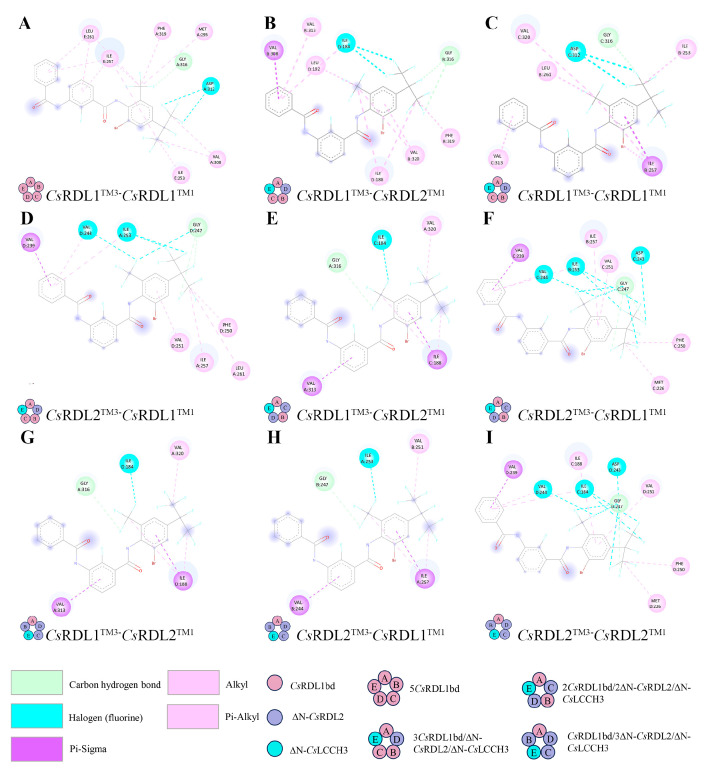
2D structures of DMBF and its interacting amino acid residues in iGABAR models. (**A**) 5*Cs*RDL1; (**B**–**D**) 3*Cs*RDL1/∆N-*Cs*RDL2/∆N-*Cs*LCCH3; (**E**,**F**) 2*Cs*RDL1/2∆N-*Cs*RDL2/∆N-*Cs*LCCH3; (**G**–**I**) *Cs*RDL1/3∆N-*Cs*RDL2/∆N-*Cs*LCCH3.

DMBF is bound to the pocket between the TM3 of *Cs*RDL1 or ∆N-*Cs*RDL2, and the TM1 of *Cs*RDL1 or ∆N-*Cs*RDL2 within iGABAR models (Figure S12). The binding affinities of DMBF to the iGABAR model assembled by *Cs*RDL1 and the models assembled by *Cs*RDL1, ∆N-*Cs*RDL2 and ∆N-*Cs*LCCH3 were −7.4 kcal/mol and −7.0 to −6.5 kcal/mol, respectively (Table 2). Furthermore, in iGABAR models assembled by *Cs*RDL1, ∆N-*Cs*RDL2 and ∆N-*Cs*LCCH3, DMBF exhibited lower binding affinities for the TM3 of ∆N-*Cs*RDL2 than for the TM3 of CsRDL1 in the iGABAR models assembled by *Cs*RDL1, ∆N-*Cs*RDL2 and ∆N-*Cs*LCCH3 at subunit ratios of 3:1:1 and 2:2:1 (Table 2). As shown in Figure 4A–I, carbon–hydrogen bonds were observed between G3’ in the TM3 of *Cs*RDL1 (G316) or ∆N-*Cs*RDL2 (G247) and DMBF. Halogen bonds were present between isoleucine (ILE), valine (VAL) or aspartic acid (ASP) residues of *Cs*RDL1 or ∆N-*Cs*RDL2 and the fluorine atoms of DMBF, while pi-alkyl and alkyl interactions were observed between *Cs*RDL1 or ∆N-*Cs*RDL2 and DMBF in homomeric and heteromeric iGABAR models. In addition, pi-sigma interactions were observed between ILE or VAL residues of *Cs*RDL1 or ∆N-*Cs*RDL2 and DMBF in heteromeric iGABAR models (Figure 4B–I).

## 4. Discussion

Alternative splicing in the Rdl subunit, e.g., Rdlbd and Rdlad, greatly increases the potential diversityof invertebrate iGABARs [1]. In this study, the *Cs*Rdl1bd transcript was obtained from the genome and transcriptome of RSB. However, the expression abundance of Rdlad is lower than that of Rdlbd [1], which explains why the transcript of *Cs*Rdl1ad cannot be obtained. Furthermore, the truncated transcripts of *Cs*Rdl2 and *Cs*Lcch3 encode proteins lacking the N-terminal extracellular region. Similarly, the ∆N-α4 subunit sequence has been identified in mouse and human [44,45]. The N-terminal domain of iGABAR subunits as a hitherto functionally unassigned region, could affect agonist potency and aligns closely with known determinants of potency in nicotinic acetylcholine receptors (nAChRs) [46]. Meanwhile, the ∆N-α4 of nAChR in Colorado potato beetle *Leptinotarsa decemlineata* is possibly a response of diverse populations to exposure of neonicotinoid insecticides [47]. It is valuable to analyze the N-terminal extracellular region role in the structure and function of nAChR [47]. Therefore, ∆N-*Cs*RDL2 and ∆N-*Cs*LCCH3 subunits is not only relevant to the structure and function of iGABARs, but also may be an adaptive response to insecticides exposure.

The Mws of various iGABARs composed of *Cs*RDL1, *Cs*RDL2 or *Cs*LCCH3 subunits were ≥440 kDa in BN-PAGE. Similarly, bands of iGABARs composed of β3 in mouse hippocampus migrated between 450 and 1236 kDa in BN-PAGE [37]. However, native iGABARs of mouse cerebellum have been reported to form two distinct complexes (~700 kDa and ~500 kDa, respectively) in BN-PAGE [35]. In the present study, the bands of iGABARs of RSB were similarly migrated to ~669 kDa and ~440 kDa, but iGABARs composed by *Cs*RDL2 formed more than two different complexes in BN-PAGE. In contrast to vertebrates, diverse native iGABARs are present in the postsynaptic membrane of RSB.

iGABARs of RSB at Mws ≥ 669 kDa are composed of post-translationally modified *Cs*RDL1, *Cs*RDL1bd, *Cs*RDL1ad, ∆N-*Cs*RDL2 and ∆N-*Cs*LCCH3. The predicted Mws of *Cs*RDL1bd, *Cs*RDL1ad (GenBank accession no. ASY91961.1), ∆N-*Cs*RDL2 and ∆N-*Cs*LCCH3 were 55 kDa, 54 kDa, 48 kDa and 37 kDa, respectively. Therefore, the two *Cs*RDL1 bands migrating at ~54 kDa and ~55 kDa correspond to *Cs*RDL1ad and *Cs*RDL1bd, respectively; the *Cs*RDL2 band migrating at ~48 kDa corresponds to ∆N-*Cs*RDL2; and the *Cs*LCCH3 band migrating at ~37 kDa corresponds to ∆N-*Cs*LCCH3. Regarding the one *Cs*RDL1 band migrating at ~70 kDa, evidence for the *N*-glycosylation of the α1, β1 and β2 iGABAR subunits has been confirmed in vertebrates [48]. Meanwhile, the phosphorylation of the β3 subunit of iGABAR is also in cultured cortical neurons [49]. Furthermore, the phosphorylation process of insect RDL is involved in the sensitivity of iGABAR to fipronil [50]. These results indicated that post-translational modifications, such as *N*-glycosylation and phosphorylation, possibly explain the increase in the Mw of native *Cs*RDL1.

*Cs*RDL1, *Cs*RDL2 and *Cs*LCCH3 composed native iGABARs in the postsynaptic membrane of RSB. *Cs*RDL1 and ∆N-*Cs*RDL2 were confirmed to be components of the native iGABARs at Mws ≥ 440 kDa. This is consistent with prior *in vitro* reconstitution studies on the RSB iGABARs. Co-injections of *Cs*Rdl1A and *Cs*Rdl2S into *Xenopus* oocytes were shown to form heteromeric iGABARs [51]. Furthermore, while *Cs*LCCH3 forms a cation-selective channel with *Cs*8916 in the RSB [52], heteromeric LCCH3/GRD channels exhibit low GABA sensitivity and insecticide pharmacology similar to homomeric RDL channels in the honeybee [53]. Meanwhile, the homomeric RDL iGABAR and potentially heteromeric RDL/LCCH3 iGABARs mediate synaptic inhibition in the antennal lobes of honeybee [54]. Likewise, ∆N-*Cs*LCCH3 was identified as a component of iGABARs at Mws ≥ 440 kDa, and full-length *Cs*LCCH3 and the post-translationally modified *Cs*RDL1 were detected in the iGABARs at Mws < 440 kDa in the present study.

*Cs*RDL1, *Cs*RDL2 and *Cs*LCCH3 were distributed in the same regions of the adult RSB head. In sections of *Drosophila* brain, anti-LCCH3 staining was confined to the cell bodies surrounding the optic neuropil and other cortical regions surrounding the central brain [55]. In contrast, *Cs*LCCH3 was distributed in the PVLP, AVLP, PED, VL, EB, LO and the periphery of the AMMC, GNG, AL and PRW. Furthermore, *Cs*RDL1 immunoreactivity was detected in the β’lobe, AVLP, LO and the periphery of the PVLP. *Cs*RDL2 was distributed in the AVLP, LO, LOP. Similarly, RDL has been observed in the β’lobe of the cricket *Acheta domesticus* L. brain [56]. Specific RDL immunostaining was evident in the LOP and LO of *Drosophila* brain [57], and intense RDL staining was observed in the LO, LOP and ventrolateral neuropils (VLP) of *Drosophila* brain sections [58]. Notably, RDL/LCCH3 do not co-localize in the same tissues of the *Drosophila* nervous system, as the ionic conductance of RDL/LCCH3 heteromeric complexes showed no significant difference compared with RDL homomers *in vitro* [55]. However, the present study demonstrated that *Cs*LCCH3, *Cs*RDL1 and *Cs*RDL2 were distributed in the AVLP, LO and the periphery of GNG, providing evidence to verify *Cs*LCCH3 probably assembling with *Cs*RDL1 and/or *Cs*RDL2 in the adult RSB head.

The assembly and intracellular transport of iGABARs in vertebrate cells have been investigated [10,59,60,61]. The intracellular assembly and proper targeting of complex heteromeric membrane proteins (e.g., iGABARs) to specialized regions including synapses, are governed by incompletely elucidated processes [10]. iGABAR’s assembly occurred within the endoplasmic reticulum (ER) and involved the interaction between chaperone molecules, immunoglobulin heavy chain binding protein and calnexin [59]. Furthermore, the N-terminal extracellular region of iGABAR subunits participate in some aspects of receptor assembly [60]. The N-terminus possibly act as a post-translational regulatory role in intracellular folding, glycosylation and assembly of the iGABAR subunits [10]. However, provided that the shorter N-terminal fragment contains an assembly-critical sequence, it will assemble with other subunits independently regardless of an additional sequence exists or is incorporated in longer N-terminal fragments [61]. Finally, assembled receptor complexes are specially targeted to appropriate sites on the membrane and stably anchored at these locations [10]. Hence, iGABARs composed of *Cs*RDL1, ∆N-*Cs*RDL2 and ∆N-*Cs*LCCH3 are likely transported to the postsynaptic membrane after intracellular assembly. Unlike intracellular subunit assembly *in vivo*, ∆N-*Cs*RDL2 and ∆N-*Cs*LCCH3 failed to express and form functional iGABAR complexes with RDL1 on the plasma membrane of *Xenopus* oocytes due to the deletion of their N-terminal signal peptides, which target newly synthesized proteins to the membrane of ER and mediate their translocation into the ER lumen [62,63]. Consequently, *in silico* analysis was implemented to explore the ligand binding characteristics of the iGABAR models assembled by *Cs*RDL1, ∆N-*Cs*RDL2 and ∆N-*Cs*LCCH3. 

DMBF was observed to bind to the pocket between the TM3 of the *Cs*RDL1 or ∆N-*Cs*RDL2 subunit and TM1 of the complementary *Cs*RDL1 or ∆N-*Cs*RDL2 subunit in iGABAR models. Similarly, in the honeybee RDL iGABAR, the pocket between the TM3 of the principal subunit and the TM1 of the complementary subunit bind abamectin, and the G3’ in the TM3 of RDL is conserved across invertebrates and represents one of the most common field-identified resistance mutations to abamectin [25]. Furthermore, knockdown of *Cs*Rdl1 and *Cs*Rdl2 *in vivo* significantly decreased the susceptibility of the RSB larvae to abamectin and fluralaner [64,65]. Fluralaner, fluxametamide and isocycloseram have been indicated to bind to the same pocket as abamectin [25]. Meanwhile, the G3’M mutation in RDL has been reported to confer resistance to broflanilide and fluralaner [24]. A hydrogen bond formed between DMBF and G277 of the *D. melanogaster* GABAR model was suggested to be the key interaction mediating DMBF antagonism by *in silico* simulations [23]. Consistent with this observation, a carbon–hydrogen bond formed between G316 of *Cs*RDL1 or G247 of ∆N-*Cs*RDL2 and DMBF was identified as the key interaction mediating the antagonism of DMBF in different iGABAR models. Additionally, DMBF exhibited higher binding affinities for heteromeric iGABAR models assembled by *Cs*RDL1, ∆N-*Cs*RDL2 and ∆N-*Cs*LCCH3 than for homomeric *Cs*RDL1 iGABAR model. Nevertheless, these *in silico* predictions are yet to be confirmed experimentally.

## 5. Conclusions

The truncated transcripts of *Cs*Rdl1 and *Cs*Lcch3 encode ∆N*-Cs*RDL2 and ∆N*-Cs*LCCH3 with Mws of 48 kDa and 37 kDa, respectively. The Mws of various native iGABARs composed of *Cs*RDL1, *Cs*RDL2 or *Cs*LCCH3 were ≥440 kDa in the postsynaptic membrane of RSB. *Cs*RDL1ad, *Cs*RDL1bd, post-translationally modified *Cs*RDL1, ∆N-*Cs*RDL2 and ∆N-*Cs*LCCH3 composed iGABARs at Mws ≥ 669 kDa. Post-translationally modified *Cs*RDL1, ∆N-*Cs*RDL2 and ∆N-*Cs*LCCH3 composed iGABARs at 440 kDa ≤ Mws < 669 kDa. Post-translationally modified *Cs*RDL1 and *Cs*LCCH3 composed iGABARs at Mws < 440 kDa. Furthermore, *Cs*LCCH3, *Cs*RDL1 and *Cs*RDL2 were distributed in the AVLP, LO and the periphery of the GNG in the adult RSB head. DMBF displayed higher binding affinities for heteromeric iGABARs assembled by *Cs*RDL1bd, ∆N-*Cs*RDL2 and ∆N-*Cs*LCCH3 than for homomeric *Cs*RDL1bd iGABAR models *in silico*. This study demonstrates that the presence of ∆N-*Cs*RDL2 and ∆N-*Cs*LCCH3 in native iGABARs of RSB provides new insights into the molecular constituents of postsynaptic ion channels and supports the rational design of novel insecticides.

## Figures and Tables

**Table 1:** Character of proteins encoded by *Cs*Rdl1, *Cs*Rdl2 and *Cs*Lcch3 transcripts.

Gene	Transcript	Protein	Number of Amino Acid (aa)	Molecular Weight (kDa)
*Cs*Rdl1bd	Csup004269.1 *	CsRDL1-2	489	55
	STRG.8675.1 *		489	55
*Cs*Rdl2	Csup004319.1	CsRDL2	424	48
	STRG.8682.1		424	48
*Cs*Lcch3	Csup009529.1 *	CsLCCH3-1	492	56
	STRG.622.1	CsLCCH3-2	329	37
	STRG.622.2	CsLCCH3-2	330	37

* the full-length transcript.

**Table 2:** Binding affinity of DMBF to iGABAR models.

iGABAR Complexes	Binding Domain	Affinity (kcal/mol)
5*Cs*RDL1	*Cs*RDL1^TM3^-*Cs*RDL1^TM1^	−7.4
3*Cs*RDL1/∆N-*Cs*RDL2/∆N-*Cs*LCCH3	*Cs*RDL1^TM3^-*Cs*RDL1^TM1^	−6.5
	*Cs*RDL1^TM3^-*Cs*RDL2^TM1^	−6.7
	*Cs*RDL2^TM3^-*Cs*RDL1^TM1^	−6.8
2*Cs*RDL1/2∆N-*Cs*RDL2/∆N-*Cs*LCCH3	*Cs*RDL1^TM3^-*Cs*RDL2^TM1^	−6.5
	*Cs*RDL2^TM3^-*Cs*RDL1^TM1^	−7.0
*Cs*RDL1/3∆N-*Cs*RDL2/∆N-*Cs*LCCH3	*Cs*RDL1^TM3^-*Cs*RDL2^TM1^	−6.6
	*Cs*RDL2^TM3^-*Cs*RDL1^TM1^	−6.5
	*Cs*RDL2^TM3^-*Cs*RDL2^TM1^	−7.0

## Data Availability

The original contributions presented in this study are included in the article/Supplementary Materials. Further inquiries can be directed to the corresponding author.

## Supplementary Materials

The following supporting information can be downloaded at: https://www.mdpi.com/article/10.3390/insects17050477/s1, Text S1:Materials and methods of peptide analysis by nanoLC-MS/MS. Figure S1: Transcript analysis of *Cs*Rdl1. Figure S2: Alignment of the amino acid sequences of *Cs*RDL1. Figure S3: Transcript analysis of *Cs*Rdl2. Figure S4: Alignment of the amino acid sequences of *Cs*RDL2. Figure S5: Transcript analysis of *Cs*Lcch3. Figure S6: Alignment of the amino acid sequences of *Cs*LCCH3. Figure S7: Separation of the postsynaptic membrane protein via BN/SDS-PAGE. Figure S8: Expression levels of *Cs*RDL1, *Cs*RDL2 and *Cs*LCCH3 in native iGABARs of different molecular weight ranges. Figure S9: Identification of *Cs*RDL2 and *Cs*LCCH3 via BN/SDS/SDS-PAGE. Figure S10: Negative control for *Cs*RDL1 (A), *Cs*RDL2 (B) and *Cs*LCCH3 (C,D). Figure S11: Ramachandran plot statistics of iGABARs assembled by various subunits. Figure S12: Binding of DMBF to the predicted structures of iGABARs assembled by various subunits. Table S1: Peptide and abundance of ∆N-*Cs*RDL2 identified by nanoLC-MS/MS. Table S2: Amino acid sequences of ∆N-*Cs*RDL2 and ∆N-*Cs*LCCH3 used in AlphaFold 3. Table S3: The overall quality factor of iGABAR models evaluated by ERRAT. Table S4: Grid center of the pocket in iGABAR models. Reference [66] was cited in the supplementary materials.

## Abbreviations

AL, antennal lobe; AMMC, antennal mechanosensory and motor center; AVLP, anterior ventrolateral neuropils; BN-PAGE, blue native PAGE; CBB R-250, Coomassie Brilliant Blue R-250; *Cs*, *Chilo suppressalis*; DMBF, desmethyl-broflanilide; EB, ellipsoid body; ER, endoplasmic reticulum; FB, fan-shaped body; GABA, γ-aminobutyric acid; GRD, GABA and glycine receptor-like subunit of *Drosophila*; GNG, gnathal ganglia; G3’, glycine at the third position; IB, Immunoblot; ICL, interior clamp; iGABARs, ionotropic γ-aminobutyric acid receptors; LAL, lateral accessory lobe; LCCH3, ligand-gated chloride channel homolog 3; LGIC, ligand-gated ion channel; LO, lobula; LOP, lobula plate; MBDL, median bundle; ME, medulla; MNG, lauryl maltose-neopentyl glycol; Mw, molecular weight; mRNA, messenger RNA; PED, pedunculus; PRW, prow; PVLP, posterior ventrolateral protocerebrum; RDL, resistant to dieldrin; RSB, rice stem borer; SAD, saddle; TM, transmembrane domain; VL, vertical lobe; WED, wedge; ∆N-CsRDL2, N-terminal-truncated CsRDL2; ∆N-CsLCCH3, N-terminal-truncated CsLCCH3; 2D, two-dimensional; 3D, three-dimensional.
