# Supplementary material for: Molecular Composition and Ligand Binding Characteristics of Native Ionotropic GABA Receptors in Rice Stem Borer, Chilo suppressalis
Source: Insects. 2026 May 6;17(5):477. doi: 10.3390/insects17050477 (PMC13207392; doi:10.3390/insects17050477)
Supplement: Supplementary file 1 [file insects-17-00477-s001.zip › insects-4225183-supplementary.pdf]

Supplementary Materials

Title

Molecular Composition and Ligand Binding Characteristics of Native Ionotropic GABA Receptors in Rice Stem Borer, *Chilo suppressalis*

The Full Names of All Authors

Enling Zhan [1], Jie Luo [1], Yuqing Zhang [1], Junyan Wang [1], Shuang Ni [2], Chunqing Zhao [1,*]

Authors Information

[1] Key Laboratory of Integrated Pest Management on Crops in East China, Ministry of Agriculture and Rural Affair; State Key Laboratory of Agricultural and Forestry Biosecurity, College of Plant Protection, Nanjing Agricultural University, Nanjing 211800, China;

[2] Plant Protection, Quarantine and Cultivated Land & Fertilizer Management Station, Huzhou Municipal Bureau of Agriculture and Rural Affairs, Huzhou 313000, China.

Corresponding Author

Chunqing Zhao – Key Laboratory of Integrated Pest Management on Crops in East China, Ministry of Agriculture and Rural Affair; State Key Laboratory of Agricultural and Forestry Biosecurity, College of Plant Protection, Nanjing Agricultural University, Nanjing 211800, China; Phone: +86-025-84399025; E-mail: zcq@njau.edu.cn; Fax: +86-025-84399063.

Authors and Email

1) Enling Zhan – eileenzhan0904@163.com;

2) Jie Luo – 2024802298@stu.njau.edu.cn;

3) Yuqing Zhang – 2364506317@qq.com;

4) Junyan Wang – 18952802515@163.com;

5) Shuang Ni – 19857183372@163.com;

6) Chunqing Zhao- zcq@njau.edu.cn.

Text S1.

Materials and methods

Peptide analysis by nanoLC-MS/MS

The sample for nanoLC-MS/MS was prepared as described previously [1]. The gel pieces were rinsed with 50 mM NH_4HCO_3, and then washed with washing buffer (50 % *v/v* 100 mM NH_4HCO_3 and 50 % *v/v* acetonitrile) for 2 times, 30 min for one time, finally used for vacuum freeze-drying after added 100 μL acetonitrile. Gel pieces were suspended in 100 mM NH_4HCO_3 (pH 8.6) containing 10 mM DTT and incubated at 56 °C for 1 h. Subsequently, 100 mM NH_4HCO_3 (pH 8.6) containing 10 mM iodoacetamide was using for alkylation at 25 °C for 45 min in the dark, and then washed with washing buffer for 2 times, 15 min for one time, finally used for vacuum freeze-drying after added acetonitrile. The dried gel pieces were transferred into 25 mM NH_4HCO_3 contained 12.5 ng/μL trypsin and incubated at 37 °C for 16 h. The supernatant was then transferred to a new tube. 50 μL of mixture (0.5% *v/v* formic acid and 20% *v/v* acetonitrile) was added into the supernatant and sonicated for 20 min, finally freeze concentrated to 15 μL and added 15 μL water prepared sample for nanoLC-MS/MS.

The protein sample were analyzed using an UltiMate 3000 RSLC nano (Thermo Fisher Scientific, Waltham, MA, USA) coupled to Orbitrap Q Exactive Mass spectrometer (Thermo Fisher Scientific). In brief, the sample were eluted from the column using a linear solvent gradient at 300 nL/min over a 120 min period, the mobile phase A is 0.1% formic acid and mobile phase B is acetonitrile contained 0.1% formic acid. The MS/MS spectra in information-dependent data acquisition operated in Xcalibur 2.1.2 software (Thermo Fisher Scientific). The MS scan was firstly record peptide spectra over the mass range of m/z 400-1,800 with 60,000 resolution ratio and followed by 27% High Energy Collision Dissociation for 10 data-dependent MS/MS scan.

Results

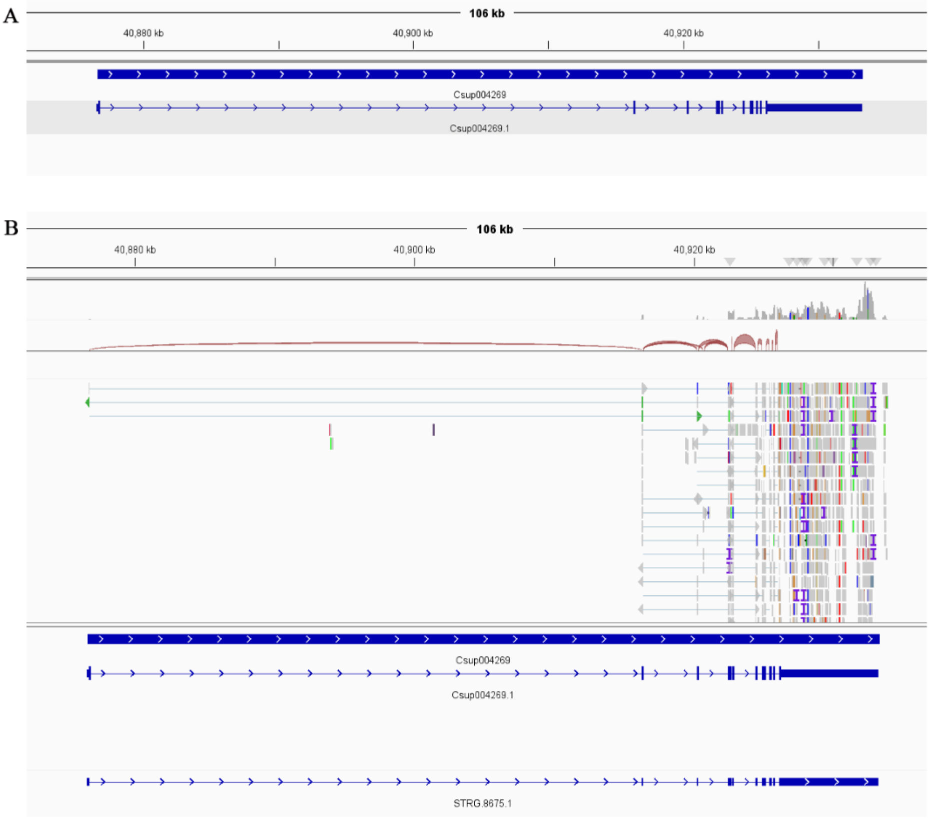

Figure S1. Transcript analysis of *Cs*Rdl1. Transcripts were blasted from the RSB genome based on the genomic GFF3 file (**A**) and the transcriptome-derived GTF file (**B**).

```
ASY91958.1_RDL1_bd              MSGARPRSAPLLLAFAAAFLPQANHVAGAGGGGMFGDVNI SAILDSFSISYDKRVRPNYGGPPVEVGVTMYVLSISSLSE   80
CSRDL1_CSUP004269.1_PROTEIN     MSGARPRSAPLLLAFAAAFLPQANHVAGAGGGGMFGDVNI SAILDSFSISYDKRVRPNYGGPPVEVGVTMYVLSISSLSE   80
CSRDL1_STRG.8675.1_PROTEIN      MSGARPRSAPLLLAFAAAFLPQANHVAGAGGGGMFGDVNI SAILDSFSISYDKRVRPNYGGPPVEVGVTMYVLSISSLSE   80
Consensus                       msgarprsaplllafaaaflpqanhvagaggggmfgdvni saildsfsisydkrvrpnyggppvevgvtmyvlsisslse
                                  N-terminal signal peptide

ASY91958.1_RDL1_bd              VKMDFTLDFYFRQFWTDPRLAYKKRTGVETLSVGSEFIKN IWVPDTFFVNEKQSYFHIATTSNEFIRIHYSGSITRSIRL  160
CSRDL1_CSUP004269.1_PROTEIN     VKMDFTLDFYFRQFWTDPRLAYKKRTGVETLSVGSEFIKN IWVPDTFFVNEKQSYFHIATTSNEFIRIHYSGSITRSIRL  160
CSRDL1_STRG.8675.1_PROTEIN      VKMDFTLDFYFRQFWTDPRLAYKKRTGVETLSVGSEFIKN IWVPDTFFVNEKQSYFHIATTSNEFIRIHYSGSITRSIRL  160
Consensus                       vkmdftldfyfrqfwtdprlaykkrtgvetlsvgsefikn iwvpdtffvnekqsyfhiattsnefirihysgsitrsirl
                                     Loop D                                       Loop A                Loop E

ASY91958.1_RDL1_bd              TITASCPMNLQYFPMDRQLCHIEIESFGYTMRDIRYKWNE GPNSVGVSSEVSLPQFKVLGHRQRAMEISLTTGNYSRLAC  240
CSRDL1_CSUP004269.1_PROTEIN     TITASCPMNLQYFPMDRQLCHIEIESFGYTMRDIRYKWNE GPNSVGVSSEVSLPQFKVLGHRQRAMEISLTTGNYSRLAC  240
CSRDL1_STRG.8675.1_PROTEIN      TITASCPMNLQYFPMDRQLCHIEIESFGYTMRDIRYKWNE GPNSVGVSSEVSLPQFKVLGHRQRAMEISLTTGNYSRLAC  240
Consensus                       titascpmnlqyfpmdrqlchieiesfgytmrdirykwne gpnsvgvssevslpqfkvlghrqrameisl ttgnysrlac
                                        cys-loop        Loop B            Loop F                       Loop C

ASY91958.1_RDL1_bd              EIQFVRSMGYYLIQIYIPSGLIVIISWVSFWLNRNATPAR VALGVTTVLTMTTLMSSTNAALPKISYVKSIDVYLGTCFV  320
CSRDL1_CSUP004269.1_PROTEIN     EIQFVRSMGYYLIQIYIPSGLIVIISWVSFWLNRNATPAR VALGVTTVLTMTTLMSSTNAALPKISYVKSIDVYLGTCFV  320
CSRDL1_STRG.8675.1_PROTEIN      EIQFVRSMGYYLIQIYIPSGLIVIISWVSFWLNRNATPAR VALGVTTVLTMTTLMSSTNAALPKISYVKSIDVYLGTCFV  320
Consensus                       eiqfvrsmgyyliqiyipsgliviiswvsfwlnrnatpar valgvttvltmttlmsstnaalpkisyvksidvylgtcfv
                                            Transmembrane region              Transmembrane region          Transmembrane

ASY91958.1_RDL1_bd              MVFASLLEYATVGYMAKRIQMRKQRFVAIQKIASEKKMPV DCPPVGDPHTLSKMGTIGRCPPGRPSVSCSEVRFKVHDPK  400
CSRDL1_CSUP004269.1_PROTEIN     MVFASLLEYATVGYMAKRIQMRKQRFVAIQKIASEKKMPV DCPPVGDPHTLSKMGTIGRCPPGRPSVSCSEVRFKVHDPK  400
CSRDL1_STRG.8675.1_PROTEIN      MVFASLLEYATVGYMAKRIQMRKQRFVAIQKIASEKKMPV DCPPVGDPHTLSKMGTIGRCPPGRPSVSCSEVRFKVHDPK  400
Consensus                       mvfaslleyatvgymakriqmrkqrfvaiqkiasekkmpv dcppvgdphtlskmgtigrcppgrpsvscsevrfkvhdpk
                                  region

ASY91958.1_RDL1_bd              AHSKGGTLENTINGGRGGAEEENPGPPPHILHPGKDISKL LGMTPSDIDKYSRIVFPVCFVCFNLMYWIIYLHVSDVVAD  480
CSRDL1_CSUP004269.1_PROTEIN     AHSKGGTLENTINGGRGGAEEENPGPPPHILHPGKDISKL LGMTPSDIDKYSRIVFPVCFVCFNLMYWIIYLHVSDVVAD  480
CSRDL1_STRG.8675.1_PROTEIN      AHSKGGTLENTINGGRGGAEEENPGPPPHILHPGKDISKL LGMTPSDIDKYSRIVFPVCFVCFNLMYWIIYLHVSDVVAD  480
Consensus                       ahskggtlentinggrggaeeenpgppphilhpgkdiskl lgmtpsdidkysrivfpvcfvcfnlmywiiylhvsdvvad
                                                                                      Transmembrane region

ASY91958.1_RDL1_bd              DLVLLEED                                         488
CSRDL1_CSUP004269.1_PROTEIN     DLVLLEED                                         488
CSRDL1_STRG.8675.1_PROTEIN      DLVLLEED                                         488
Consensus                       dlvlleed
```

Figure S2. Alignment of the amino acid sequences of *Cs*RDL1.

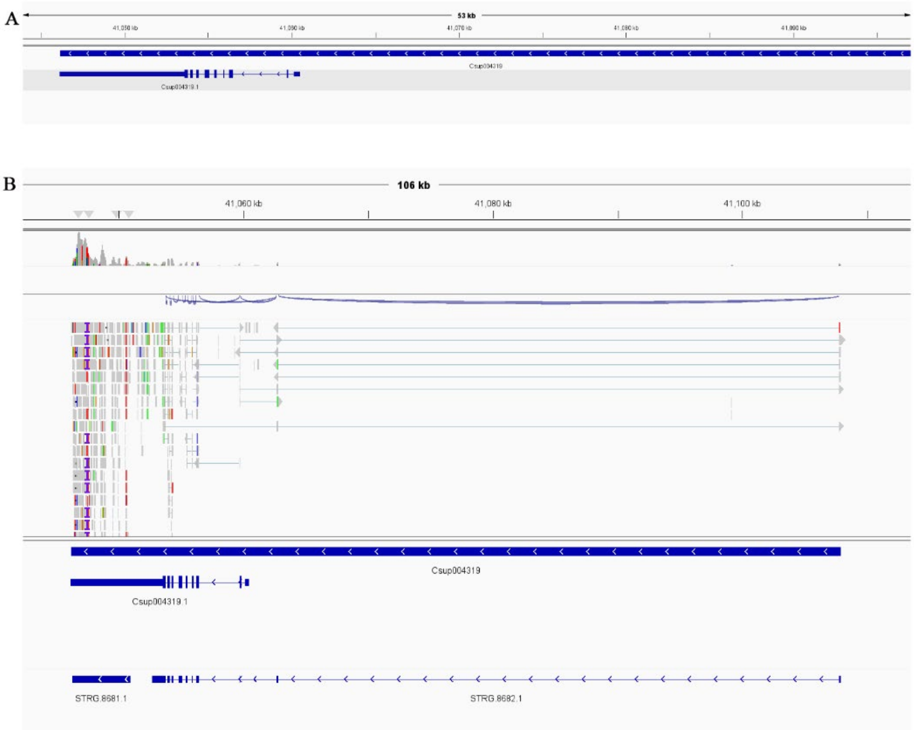

Figure S3. Transcript analysis of *Cs*Rdl2. Transcripts were blasted from the RSB genome based on the genomic GFF3 file (**A**) and the transcriptome-derived GTF file (**B**).

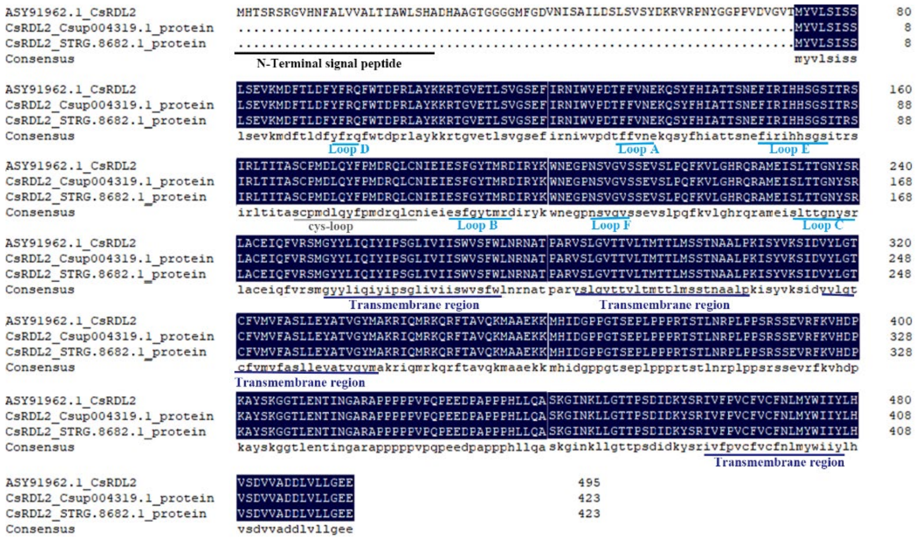

Figure S4. Alignment of the amino acid sequences of *Cs*RDL2.

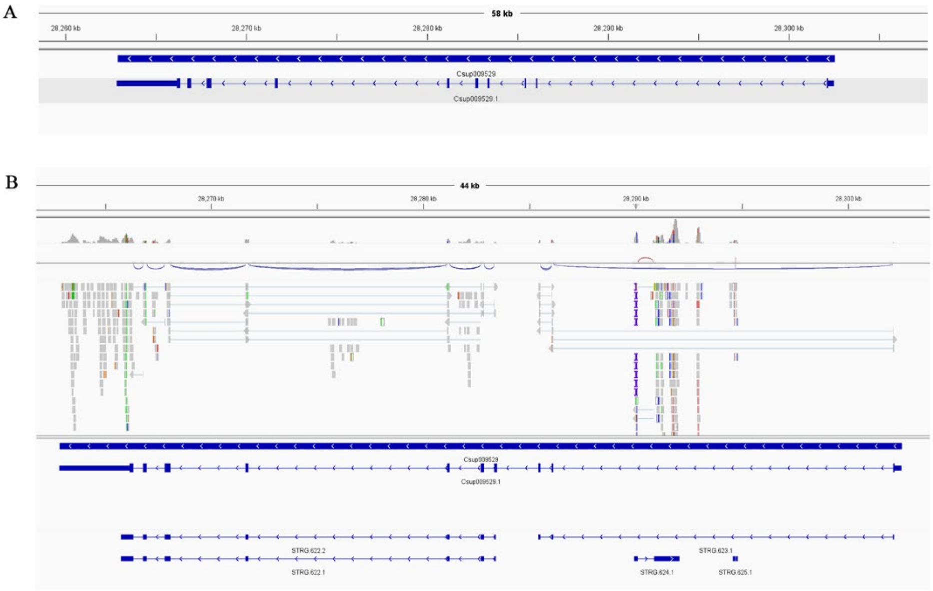

Figure S5. Transcript analysis of *Cs*Lcch3. Transcripts were blasted from the RSB genome based on the genomic GFF3 file (**A**) and the transcriptome-derived GTF file (**B**).

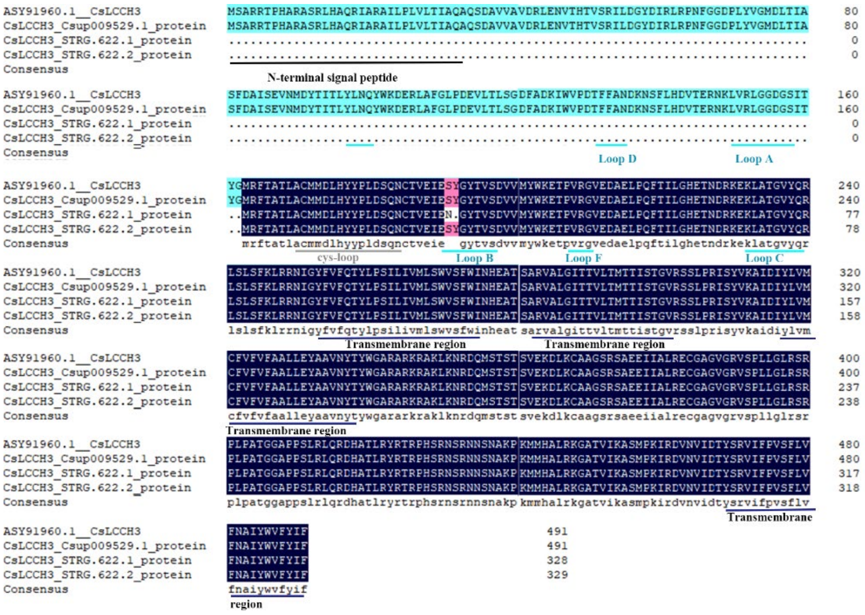

Figure S6. Alignment of the amino acid sequences of *Cs*LCCH3.

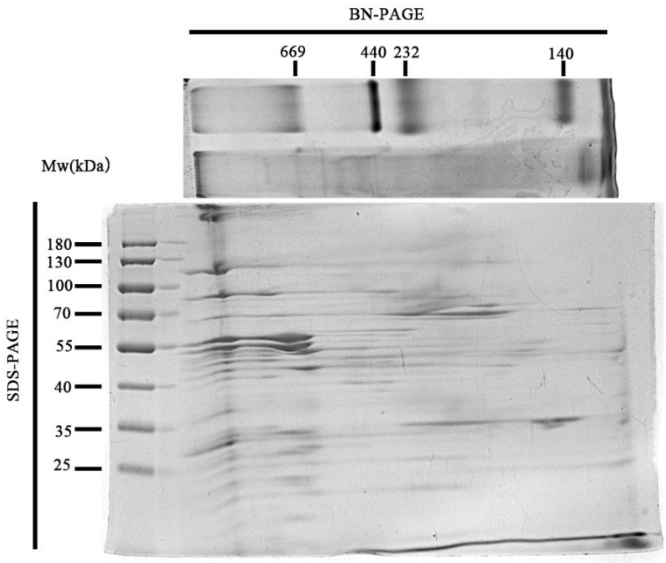

Figure S7. Separation of the postsynaptic membrane proteins via BN/SDS-PAGE. Proteins were stained with CBB R-250 staining.

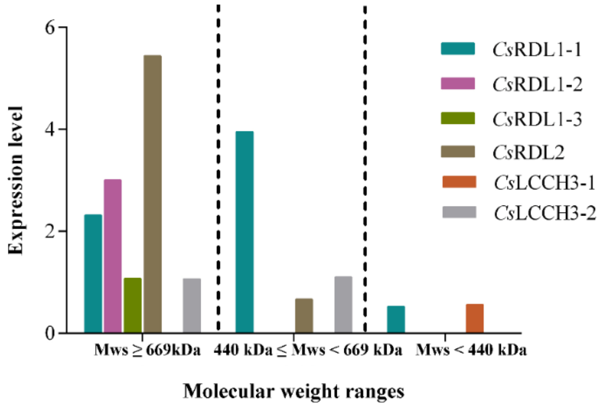

Figure S8. Expression levels of *Cs*RDL1, *Cs*RDL2 and *Cs*LCCH3 in native iGABARs of different molecular weight ranges.

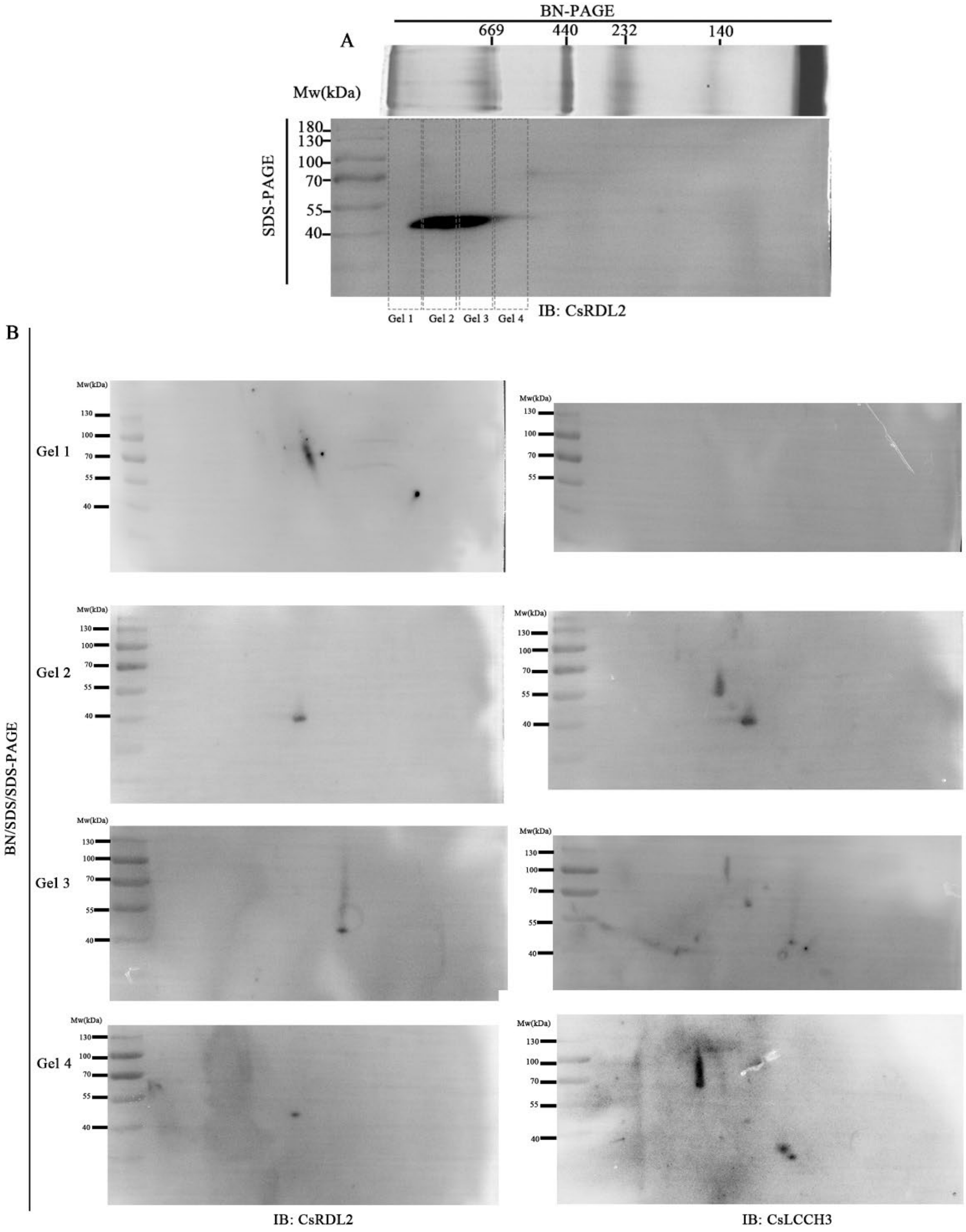

Figure S9. Identification of *Cs*RDL2 and *Cs*LCCH3 via BN/SDS/SDS-PAGE. (**A**) The BN/SDS-PAGE gel lanes containing *Cs*RDL2 were cut into individual gel lanes; (**B**) Proteins separated via BN/SDS/SDS-PAGE and identified with antibodies against *Cs*RDL2 and *Cs*LCCH3, respectively.

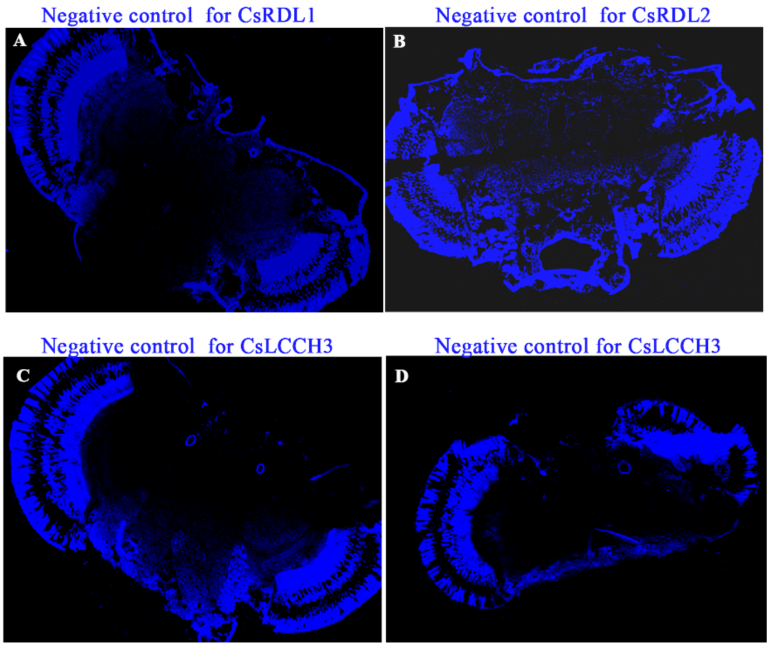

Figure S10. Negative control for *Cs*RDL1 (**A**), *Cs*RDL2 (**B**) and *Cs*LCCH3 (**C, D**). Rabbit pre-immune serum used as a primary antibody in the negative control. The blue channel corresponds to signals from the negative control.

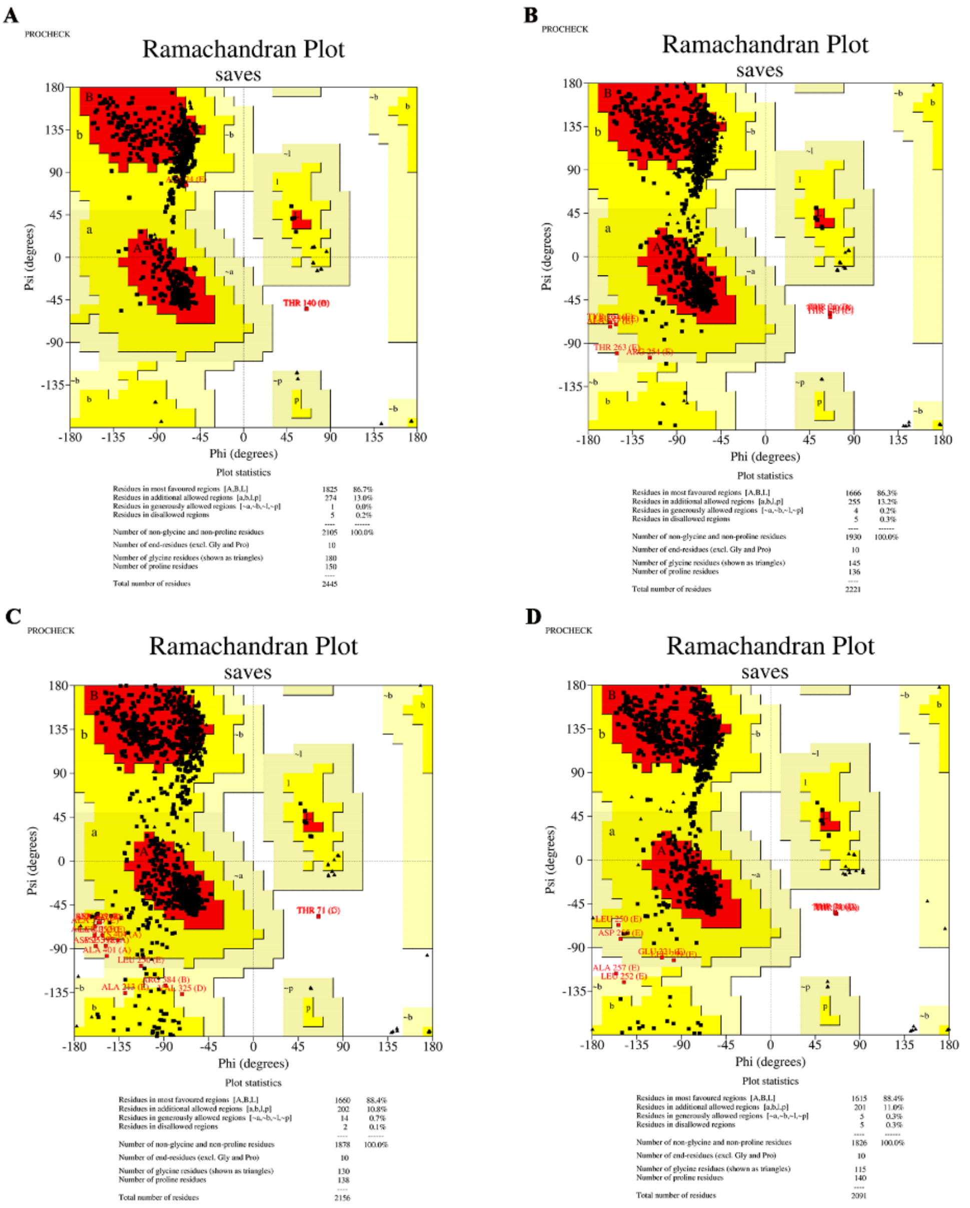

Figure S11. Ramachandran plot statistics of iGABARs assembled by various subunits. (**A**) 5*Cs*RDL1; (**B**) 3*Cs*RDL1/ΔN-*Cs*RDL2/ΔN-*Cs*LCCH3; (**C**) 2*Cs*RDL1/2ΔN-*Cs*RDL2/ΔN-*Cs*LCCH3; (**D**) *Cs*RDL1/3ΔN-*Cs*RDL2/ΔN-*Cs*LCCH3.

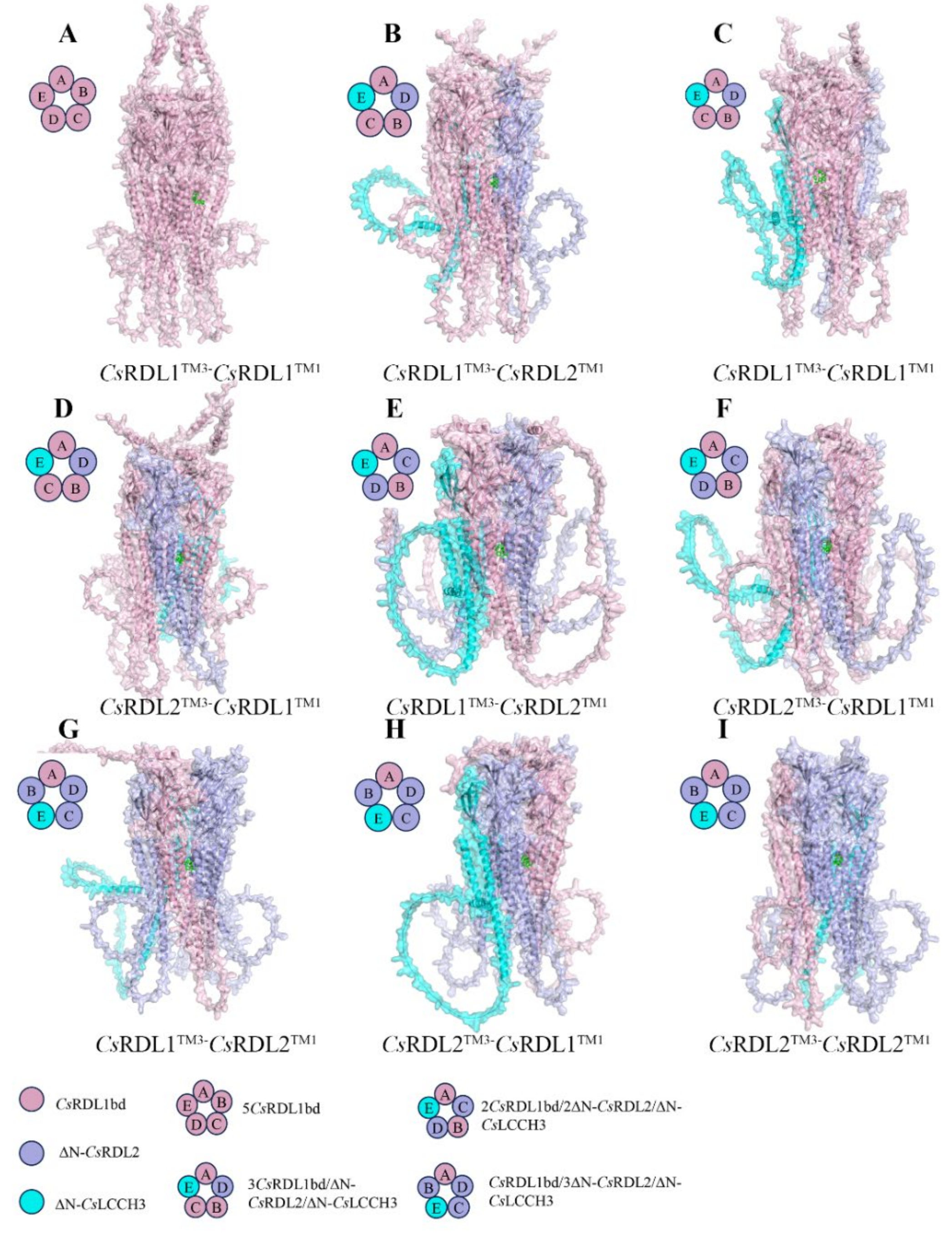

Figure S12. Binding of DMBF to the predicted structures of iGABAR models assembled by various subunits. (**A**) 5*Cs*RDL1; (**B**–**D**) 3*Cs*RDL1/ΔN-*Cs*RDL2/ΔN-*Cs*LCCH3; (**E, F**) 2*Cs*RDL1/2ΔN-*Cs*RDL2/ΔN-*Cs*LCCH3; (**G**–**I**) *Cs*RDL1/3ΔN-*Cs*RDL2/ΔN-*Cs*LCCH3.

Table S1. Peptide and abundance of ΔN-*Cs*RDL2 identified by nanoLC-MS/MS.

	N-terminal Truncated *Cs*RDL2	Abundance
1	MYVLSISSLSEVKMDFTLDFYFRQFWTDPRLAYKKRTGVETLSVGSEFIR	
51	NIWVPDTFFVNEKQSYFHIATTSNEFIRIHHSGSITRSIRLTITASCPMD	
101	LQYFPMDRQLCNIEIESFGYTMRDIRYKWNEGPNSVGVSSEVSLPQFKVL	
151	GHRQRAMEISLTTGNYSRLACEIQFVRSMGYYLIQIYIPSGLIVIISWVS	
201	FWLNRNATPARVSLGVTTVLTMTTLMSSTNAALPKISYVKSIDVYLGTCF	19207
251	VMVFASLLEYATVGYMAKRIQMRKQRFTAVQKMAAEKKMHIDGPPGTSEPL	
301	PPPRTSTLNRPLPPSRSSEVRFKVHDPKAYSKGGTLENTINGARAPPPP	
351	PVPQPEEDPAPPPHLLQASKGINKLLGTTPSDIDKYSRIVFPVCFVCFNL	
401	MYWIIYLHVSDVVADDLVLLGEEN	

The identified peptide is highlighted in red.

Table S2. Amino acid sequences of ΔN-*Cs*RDL2 and ΔN-*Cs*LCCH3 used in AlphaFold 3.

Protein	Amino Acid Sequences
ΔN-*Cs*RDL2	MYVLSISSLSEVKMDFTLDFYFRQFWTDPRLAYKKRTGVETLSVGSEFI<br>RNIWVPDTFFVNEKQSYFHIATTSNEFIRIHHSGSITRSIRLTITASCPM<br>DLQYFPMDRQLCNIEIESFGYTMRDIRYKWNEGPNSVGVSSEVSLPQ<br>FKVLGHRQRAMEISLTTGNYSRLACEIQFVRSMGYYLIQIYIPSGLIVII<br>SWVSFWLNRNATPARVSLGVTTVLTMTTLMSSTNAALPKISYVKSID<br>VYLGTCFVMVFASLLEYATVGYMAKRIQMRKQRFTAVQKMAAEKK<br>MHIDGPPGTSEPLPPPRTSTLNRPLPPSRSSEVRFKVHDPKAYSKGGTL<br>ENTINGARAPPPPPVPQPEEDPAPPPHLLQASKGINKLLGTTPSDIDK<br>YSRIVFPVCFVCFNLMYWIIYLHVSDVVADDLVLLGEEN
ΔN-*Cs*LCCH3	MRFTATLACMMDLHYYPLDSQNCTVEIESYGYTVSDVVMYWKETPV<br>RGVEDAELPQFTILGHETNDRKEKLATGVYQRLSLSFKLRRNIGYFVF<br>QTYLPSILIVMLSWVSFWINHEATSARVALGITTVLTMTTISTGVRSSLP<br>RISYVKAIDIYLVMCFVFVFAALLEYAAVNYTYWGARARKRAKLKNR<br>DQMSTSTSVEKDLKCAAGSRSAEEIIALRECGAGVGRVSPLLGLRSRP<br>LPATGGAPPSLRLQRDHATLRYRTRPHSRNSRNNSNAKPKMMHAL<br>RKGATVIKASMPKIRDVNVIDTYSRVIFPVSFLVFNAIYWVFYIFD

Table S3. The overall quality factor of iGABAR models evaluated by ERRAT.

iGABAR Complexes	Overall Quality Factor
5*Cs*RDL1	88.145
3*Cs*RDL1/ΔN-*Cs*RDL2/ΔN-*Cs*LCCH3	81.638
2*Cs*RDL1/2ΔN-*Cs*RDL2/ΔN-*Cs*LCCH3	84.760
*Cs*RDL1/3ΔN-*Cs*RDL2/ΔN-*Cs*LCCH3	83.098

Table S4. Grid center of the pocket in iGABAR models.

iGABAR Complexes	Binding Domain	Grid Center (x, y, z)
5*Cs*RDL1	*Cs*RDL1^{TM3}-*Cs*RDL1^{TM1}	1.246, -15.465, -19.9
3*Cs*RDL1/ΔN-*Cs*RDL2/ΔN-*Cs*LCCH3	*Cs*RDL1^{TM3}-*Cs*RDL1^{TM1}	9.99, 19.749, -2.623
	*Cs*RDL1^{TM3}-*Cs*RDL2^{TM1}	8.123, 17.875, -5.052
	*Cs*RDL2^{TM3}-*Cs*RDL1^{TM1}	0.256, 7.052, -20.407
2*Cs*RDL1/2ΔN-*Cs*RDL2/ΔN-*Cs*LCCH3	*Cs*RDL1^{TM3}-*Cs*RDL2^{TM1}	16.884, -8.57, -7.181
	*Cs*RDL2^{TM3}-*Cs*RDL1^{TM1}	-3.6, -8.716, -17.09
*Cs*RDL1/3ΔN-*Cs*RDL2/ΔN-*Cs*LCCH3	*Cs*RDL1^{TM3}-*Cs*RDL2^{TM1}	13.138, -0.13, -12.658
	*Cs*RDL2^{TM3}-*Cs*RDL1^{TM1}	13.38, -11.017, 9.696
	*Cs*RDL2^{TM3}-*Cs*RDL2^{TM1}	-7.611, 3.186, -16.934

References

1 Rappsilber, J.; Mann, M.; Ishihama, Y. Protocol for micro-purification, enrichment, pre-fractionation and storage of peptides for proteomics using StageTips. Nat Protoc, 2007, 8, 1896-1906. https://doi.org/10.1038/nprot.2007.261.
